# Historical Evolution of Skin Grafting—A Journey through Time

**DOI:** 10.3390/medicina57040348

**Published:** 2021-04-05

**Authors:** Michael Kohlhauser, Hanna Luze, Sebastian Philipp Nischwitz, Lars Peter Kamolz

**Affiliations:** 1 COREMED—Cooperative Centre for Regenerative Medicine, Joanneum Research Forschungsgesellschaft mbH, 8010 Graz, Austria; hanna.luze@joanneum.at (H.L.); sebastian.nischwitz@joanneum.at (S.P.N.); lars.kamolz@medunigraz.at (L.P.K.); 2 Division of Plastic, Aesthetic and Reconstructive Surgery, Department of Surgery, Medical University of Graz, 8036 Graz, Austria

**Keywords:** skin grafting, skin transplantation, skin substitutes, history, burns

## Abstract

Autologous skin grafting was developed more than 3500 years ago. Several approaches and techniques have been discovered and established in burn care since then. Great achievements were made during the 19th and 20th century. Many of these techniques are still part of the surgical burn care. Today, autologous skin grafting is still considered to be the gold standard for burn wound coverage. The present paper gives an overview about the evolution of skin grafting and its usage in burn care nowadays.

## 1. Introduction

The skin is not only the largest organ of the human body but also the first line of defense against harmful influences such as mechanical forces, microorganisms, or radiation. It maintains thermoregulation and fluid balance as well as acts as a sensory organ that is able to register pressure, temperature, and pain, due to specific receptors. The integrity of human skin plays an essential role in maintaining physiological homeostasis of the body. A large skin loss caused by e.g., burns, can cause a disturbance of this integrity [1,2]. To date, autologous skin grafting is commonly considered as the gold standard for the coverage of large skin defects. While the usage of meshed split thickness skin grafting is the best option for the treatment of extensive burns, unmeshed sheet grafting is used for small burns and in aesthetically important regions. Full-thickness skin grafting achieves the best aesthetic and functional results in burn injury reconstruction [3,4,5].

The origin of skin grafting can be traced back for more than 3500 years. Many techniques and adjustments have been established over time. This article gives an overview about the historical evolution of skin grafting, including the development of common techniques, and further explains their usage in burn care nowadays. In addition, the development and the usage of further established techniques are presented. Finally, the split-thickness skin graft associated donor site problems are analyzed, and solutions are discussed.

## 2. The Origin of Skin Grafting

Skin grafting was already practiced in the Egyptian Empire. This ancient skin graft technique was already taking place 1500 before Christ (BC) and was documented in an old papyrus role called “Ebert papyrus” [6]. According to the “Sushruta Samhita”, one of the early texts of Ayurveda, skin grafting was also performed by the ancient Hindu more than 3000 years ago. Members of the Koomas Caste used subcutaneous fat and skin from the gluteal region as free skin graft [6,7,8]. In the 1st and 2nd century, Celsus and Galen used skin grafts to treat facial defects. Furthermore, Celsus developed a method to reconstruct the foreskin of Jewish men in historical Roman Empire [7]. During a long period, most of the knowledge was forgotten. For example, the earlier known techniques of free skin grafting got lost in the Middle Ages. The method seemed to be forgotten until the early 19th century. In 1804, Giuseppe Baroni demonstrated successful transplantations of free skin grafts in ram. Seventeen years later, the first verified successful nasal reconstruction by usage of free skin autografting was performed by Professor Bünger, which was inspired by the ancient Indian method [8,9,10].

In 1869, Jaques-Louis Reverdin, a Swiss surgeon, presented a successful experiment of free skin grafting [6,7,9,11]. By using the tip of a lancet, Reverdin harvested epidermal small bits from the arm of the patient and fixed them into the middle of wound with a diachylon bandage. Although the procedure is known as “Pinch Graft” today, Reverdin called his technique “Epidermic Grafting” [6,7]. In May 1870, Georg David Pollock was the first surgeon who performed a successful pinch graft in a burn victim case. His patient was an 8-year-old girl who suffered a large defect of her right thigh by severe burns [6,7,12,13]. Pollock transplanted two small pieces from the abdomen to the middle of the lower part of the defect. Three weeks later, a second series was transplanted into the wound. After 6 weeks, all grafts grew well and divided the defect into two parts. Two further pinch grafts, harvested from the abdomen, were successfully transplanted. Pollock also performed the first known allografts to a burned patient in combination with autologous pinch grafts. However, the allogeneic grafts did not grow and were gradually destroyed, but they seemed to stimulate the spontaneous healing of the autologous pinch grafts [12,13].

## 3. The Split-Thickness Skin Graft

The history of split-thickness skin grafting dates back to the late 19th century. The earliest known split-thickness skin grafting method was developed by Ollier in 1872. His results revealed not only a faster healing but also less scar formation and therefore less scar contractures by covering the whole wound surface with skin grafts. Since these grafts included epidermis as well parts of the dermis, Ollier called his technique “dermo-epidermic grafting” [6]. Prof. Carl Thiersch, chairman of the surgery department in Leipzig, presented his technique at the 15th Congress of German Surgical Association in 1886 [6,14]. His technique advised to cut the skin with a razor blade as thin as possible via sharp horizontal incisions to produce thin strips of epidermis, only including small parts of dermis [6,7,15]. Thiersch’s technique obtained national publicity, which is known as “Thiersch Graft” [6]. Caused by the similarity of both discoveries, the method is also known as “Ollier–Thiersch graft”. In 1929, Blair and Brown presented their method of “split skin grafts” of intermediate thickness. These grafts differ from “Ollier–Thiersch graft” in regard of the thickness due to included layers of dermis. While Ollier and Thiersch advised to include only little more than the epithelial layer, the split skin grafts of intermediate thickness also included an appreciable amount of the dermal layer. The idea was to preserve the advantages of both, the “Ollier–Thiersch graft”, as well those of the full-thickness skin graft [16]. In 1941, Earl C. Padgett, an American surgeon, developed a new method of split-thickness skin grafting by using a manual dermatome. The “three-quarter”-thickness skin graft demonstrated good graft take, and the dermatome enabled the possibility of new skin donor sites, which were not available by free hand skin grafting methods [17].

**Figure 1:**
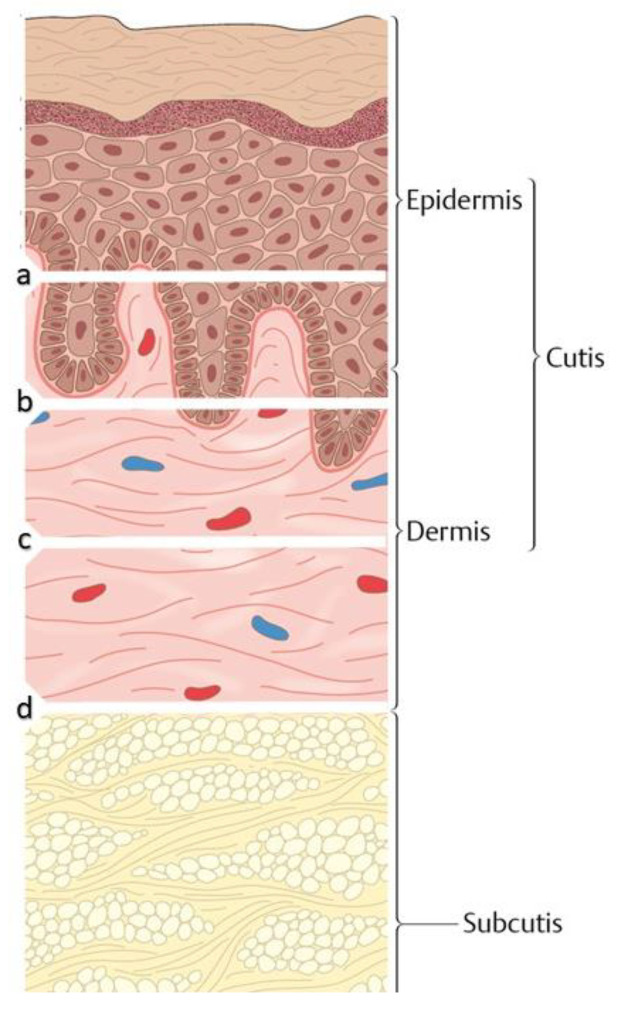
The classification of split thickness skin grafts (**a**–**c**) and full thickness skin graft (**d**) according to the thickness of the layer. a = 0.15–0.3 mm; b = 0.3–0.45 mm; c = 0.5–0.6 mm; d > 0.6 mm.

The current STSG classification is based according to their thickness into thin STSGs (0.15 to 0.3 mm), intermediate STSGs (0.3 to 0.45 mm), and thick STSGs (0.45 to 0.6 mm) [18]. The different thickness layers are displayed in Figure 1.

In 1970, Janzekovic demonstrated her concept of early excision and wound coverage with autologous split-thickness skin grafts [19]. This method is considered as the current gold standard in surgical burn treatment, even today [4,5]. A major advantage of split-thickness skin grafting is the possibility of using the same donor site repeatedly after healing, which typically occurs within 7–14 days [20,21]. Further benefits are less morbidity and less scar formation in donor sites, which increase the contemplable donor sites, compared with full-thickness skin grafts [22]. Commonly used donor sites are thighs, legs, abdomen, back, arms, forearms, and chest [22,23]. In extensive burns, with a lack of eligible skin, the scalp or even scrotum can be used as last resort donor sites [24,25,26,27]. A distinction must be made between unmeshed STSGs (sheet grafts) and STSGs extended by specifically expanding methods. Sheet grafts are commonly used for small burns, while meshed split-thickness skin grafting depicts the best alternative for the coverage of large burns [23,28,29].

### 3.1. Sheet Graft

Sheet grafting is considered to be the gold standard for the treatment of small burns and to cover sensitive areas [5,28,30]. Sheet grafts accelerate the end of the inflammatory phase and offer a better vascularization and re-innervation. Further benefits are the lower tendency for scar formation and contractures, a better aesthetic outcome, and no permanent mesh pattern in contrast to meshed skin grafts. Therefore, sheet grafts are appropriate to cover visible and functionally important areas [28,30]. Sheet grafts can as well be used to cover aesthetic and functional important areas in severe burns, in order to save donor sites instead of initial coverage with full-thickness skin grafts [31]. The disadvantages of sheet grafting are the need of larger donor sites, the risk of hematoma formation, the danger of losing the graft because of its impermeability, and the inability to cover severe burns, which is caused by a lack of donor sites [28].

### 3.2. Mesh Graft

Professor Otto Lanz was dissatisfied with the fact that the donor site of a Thiersch graft was still an open wound, while the initial defect already healed. He investigated various methods, but none of the experiments led to a satisfactory result. Due to a childhood game, which was used to build a paper accordion, he was encouraged to use the same method for skin grafting. Lanz developed an accordion-like expansion of a Thiersch graft in 1908, which not only served the purpose of covering the defect but also the newly formed wound of the donor site [32].

In 1964, James C. Tanner, a plastic surgeon at the Long Memorial Hospital, Atlanta developed a new method to produce expanded STSGs by usage of a new device named “Tanner–Vandeput mesh dermatome”. By rolling split-thickness skin grafts through the novel dermatome, the machine cuts the skin grafts into a mesh with ribbons of skin 0.050-inches wide. Tanner’s mesh grafting enabled skin grafts expanding up to a ratio 1:3, reducing the area of the donor site and offering the possibility of covering more wound area [33]. Nowadays, ratios up to 1:6 or even 1:9 are possible by using special devices [23]. Complete wound coverage can be achieved in approximately 10 days through rapid epithelialization in the absence of infection. Furthermore, a drainage of exudate and hemorrhage, as well regaining of areas lost by shrink is possible, due to the mesh-like structures. These properties are ideal for the treatment of burn injuries and large defects with limited donor sites [34,35]. The advantage of fluid drainage and a similar cosmetic outcome as with sheet grafts is achievable with a meshing ratio of 1:1 [28]. Additionally, mesh grafting shows a high percentage of graft take and enables covering large wound areas. Further benefits are the decrease of operating time and the reduction of the required number of necessary surgeries to achieve full rehabilitation. 

Due to the advantages described above, the mesh skin graft method is well established and is considered as the standard method to surface large areas in severe burn treatment, even today [4,5,23,36].

### 3.3. Meek Technique

In 1958 Cicero Parker Meek, a general practitioner at the Aiken Country Hospital in South Carolina known for his great interest in burn care, published an article called “Successful microdermagrafing using the Meek–Wall microdermatome”. The Meek–Wall microdermatome consisted of 13 blades driven by an electronic engine. Flat cork plates served as carriers for the skin grafts [37,38]. The functions were described by a case report of a 14-year-old burn victim with 25% total body surface area. Meek cut conventional split-thickness skin grafts (0.0125 inches) into units 1/16 inches square (40 mm^2^). Subsequently, the microdermagrafts were saturated in plasma and evenly distributed to prefold parachute silk bandages, which were placed directly on the wound. After ten days, the grafted areas could be left exposed [37]. Meek’s microdermal grafting enabled the possibility of covering large denuded areas after severe burns successfully by widely expanded stamp autografts [39]. In 1965, Meek published another article to describe his method step by step in detail and reported about the experience he made with the Meek–Wall dermatome [40]. The Meek technique was slowly forgotten after the development of the mesh grafting technique by Tanner in 1964. In the early 1990s, Meek’s technology was rediscovered and improved by Dutch surgeons at the burn unit of the Red Cross Hospital in Beverwijk. The Meek technique was used successfully in the treatment of severe burns, when insufficient suitable donor sites were available for wound coverage with mesh grafts only. The clinical results of this modified Meek technique were first published by Kreis et al. in 1993 [41]. Thenceforth, the Meek technique returned in the clinical setting. The usage of the Meek technique offers many advantages, especially in the treatment of severe burns, which are often affected by the lack of donor sites. The Meek technique enabled an expansion of surface area coverage from 1:3 up to 1:9. The micro grafts allow a shorter duration and more uniform epithelialization than other techniques. Another benefit is the easy application compared to the difficult handling of higher expanded mesh grafts (1:6 or 1:9) [42,43,44,45,46]. In shortage of donor sites, Meek enabled the expansion of smaller grafts, as well the usage of donor sites, which cannot be grabbed by other grafting techniques [46,47]. Furthermore, the re-epithelialization time seems to be shorter with the Meek technique compared to mesh grafts [43]. Some graft takes failed by contamination, but these were mostly restricted to a partial area without affecting nearby skin islands [42,48], while the observed total take rate with Meek grafts was described by several authors between 82.3% and 90% [42,44,45,48,49,50]. Major disadvantages of Meek’s method are the protracted procedure as well the necessity of more staff in the operating theater [42,45].

## 4. The Full-Thickness Skin Graft

If the skin graft includes the entire thickness of the dermis, the appropriate term is full-thickness skin graft (FTSG) [51,52]. Full-thickness skin grafts are not widely used for emergency burn care but are ideal for the reconstruction after initial treatment and for scar corrections [31,53].

The first known full-thickness skin graft technique was presented by John Reissberg Wolfe in 1875. Wolfe described the correction of an ectropium by using a full-thickness skin graft after cutting accurately to the shape of the defect. A similar technique was described by Georg Lawson in 1870 and 1871, even though his name is not associated with the development of the full-thickness skin graft [7,54,55]. Fedor Krause, a German surgeon based in Hamburg, established the usage of full-thickness skin grafts. At the XXIII Congress of the German Surgical Association in 1893, Krause advised the usage of the Wolfe graft for all cases, where the Thiersch graft showed unsatisfactory outcomes and reported 21 cases in which full-thickness skin grafts were successfully transplanted. Krause’s knowledge was well received not only nationally but also internationally, and the full-thickness graft achieved great popularity [6].

Nowadays, FTSGs are considered to achieve best results in burn deformity reconstructions due to less scar formation, good plasticity, elasticity, mobility, and aesthetic outcome, as well due to providing improved texture and color matching. After a long-term period, full-thickness skin grafts are almost similar to intact skin. Especially facial and palmar burns are one of the most challenging problems in burn injury treatment, but they are also a burden to the individual’s psychological sentiment by functional disorders, structural defects, scars formation, scar contractures, as well soft tissue or hard tissue defects [56,57]. Hand burns often affect children in particular, leading to scar contracture and resulting in functional implications and disturbed evolution of the child’s hand [22]. Full-thickness skin grafts are able to achieve excellent results in reconstruction, are aesthetically as well functionally superior to those obtained from sheet grafts, and therefore are the ideal choice for treatment of facial and palmar defects and scars suffered by burns [53,56,57,58]. Despite this occurrence of excellent results, various influencing factors must be observed by the performing surgeon at the selection of the eligible donor site: skin quality, skin color, texture, damage due to ultraviolet radiation, skin thickness, convenience, size, possibility of contractility, and scar formation after graft taking, as well the fact that donor sites can only be harvested once [53]. Summarizing, the usage of FTSGs achieves better aesthetic and functional results compared with STSGs. However, FTSGs have various limitations; thus, their usage should be reserved for the reconstruction of late deformities, especially in sensitive areas.

## 5. Allogeneic and Xenogeneic Transplants

The earliest proposals using foreign human skin or animal skin for burn injuries date back to the 19th century, but these failed [12,13,59,60,61]. Nevertheless, the first successes occurred not until the middle of the 20th century [31,62]. Even these days, allografts, usually taken by cadaver and xenografts, mostly from porcine skin, are used as temporary skin substitutes ahead to final coverage with autologous skin grafting, which provides a temporary coverage for up to 14 days, followed by the immunoreaction and rejection [5,63]. Currently, two common types of foreign skin are available, cryo-preserved or glycerol-preserved allografts [64]. Both preservation procedures have shown different benefits. While cryo-preserved allografts demonstrated a better tissue viability, the glycerol preservation is cost-efficient, can reduce the antigenicity, and leads to longer storage periods [65,66,67,68]. 

The usage of porcine skin as a temporary wound dressing became popular in the 1960s, and it is still the most commonly used xenograft [62]. The major advantages of porcine skin include its easy availability and histopathological similarity to human skin [69,70]. Novel approaches are the usage of fish skin as a temporary biological dressing [71,72,73]. Alam and Jeffery demonstrated complete re-epithelialization in the absence of infection or adverse reaction in a case series of 10 patients with partial thickness burns treated with fish skin [72]. Bruno et al. presented Nile Tilapia fish skin as an easily available and cost-effective option as a xenograft [73]. A phase II randomized controlled trial showed that patients treated with Nile Tilapia fish skin had a statistically significant reduction of the mean time for re-epithelialization, significant pain reduction, and lower requirement of dressing changes compared with patients under silver sulfadiazine treatment [71]. According to these findings, fish skin seems to be a promising candidate as an effective and low-cost biological dressing for burn treatment, especially in middle- to low-income countries. However, for the introduction into the hospital setting, further investigations need to be done.

### Sandwich Technique

Another option for the treatment of extensive loss of skin is the combined application of autologous STSGs and allografts. In 1981, Alexander et al. first publicized the successful usage of widely meshed autologous split-thickness skin grafts (1:6), which were overlayed by meshed allogeneic skin grafts (ratio 1:2) [74]. Although the usage of widely expanded mesh grafts has shown a bad outcome, the dressing with allografts enabled this method for the treatment of extensive burns, despite the lack of donor sites [74,75,76]. Good clinical results and a high rate of re-epithelization were achieved with the sandwich technique [74,77].

## 6. A Brief History of Skin Substitutes and Their Use Today

Despite the great achievements of the expansion methods of Tanner and Meek, the treatment of extensive burns still presents crucial problems. Due to insufficient amounts of healthy native skin during the acute phase, an initial coverage with autologous skin is sometimes not possible. Therefore, several methods were developed over the past years to handle the absence of donor sites.

### 6.1. Cell Cultures

The first published formation of epidermis-like tissue by in vitro cultivation of human epidermal keratinocytes was performed by Rheinwald and Green in 1975 [78]. Ten years later, the first usage of human cultured epidermal autografts (CEAs) in a clinical case series were performed in 1980 by Connor et al [79]. Nowadays, the usage of CEAs is reserved for extensive burn injuries as a last resort opportunity when significantly less donor sites are remaining and other alternatives are not applicable. However, the application of CEAs is extremely time-consuming; these grafts are very fragile and susceptible to shear forces, and they additionally have a higher rate of blistering and re-grafting requirement [80,81]. A novel approach in the wound closure of extensive burned patients is the conjunction of CEAs with split-thickness skin grafting using high expansion techniques [82,83,84]. These methods enable the coverage of fragile body parts susceptible to pressure or shear forces with autologous STSGs, while insensitive areas can be covered with CEAs [82]. Another benefit is the reduction of donor sites [82,84]. Since 2007, cell therapies such as CEAs are considered as “Advanced Therapy Medicinal Products” (ATMP) by European Directives along with associated Regulations by the European Parliament. The aim of this adaption was to improve the safety and efficiency of cell therapy by standardization. Due to these new regulations, cell cultures need to be accomplished in an approved laboratory, along with new quality inspection measures and complex pathways, which the medical practitioner has to assess before deciding if a technique should be introduced [85]. Furthermore, the compliance of Good Manufacturing Practice requirements, as well the marketing authorization, for cell therapy production in hospital settings leads to higher costs [86]. Although it is important to ensure the safety for burn patients, these changes present a major challenge for clinical research not only in burn care but also in other cell research facilities in the hospital settings [85]. Gardien et al. showed the possibility of conducting a multicenter clinical trial that follows all requirements consistent with the ATMP guidelines [87].

### 6.2. Dermal Substitutes

In the last decades, burn care research has shifted from pure survival to a better quality of survival by focusing on improvement of the scars outcome and contractures prevention. Better functional and aesthetical results can be achieved through the use of dermal substitutes during the acute phase of burns [88]. Therefore the gain in importance of alloplastic or mixed synthetic–biological carriers with different alloplastic materials as dermal substitutes were observed in recent decades.

The first dermal analoga called “Integra^®^” was developed in the 1980s by Yannas and Burke as an alternative burn injury treatment [89,90]. Integra^®^ consists of a dermal layer of bovine collagen and chondroitin-6-sulfate glycos-aminoglycan (GAG), as well an epidermal layer of silicone, and it was designed for the treatment of fresh excised full-thickness burns. The silicone layer has to be removed and replaced by a split-thickness skin graft after 2 or 3 weeks [91,92]. Aesthetic and functional results similar to healthy skin can be achieved in burned hands treated with Integra^®^ and STSGs [93,94]. Currently, Integra^®^ represents the most accepted artificial skin substitute due to favorable long-term use and outcomes [88,91,92]. Recently, a single layer version of Integra^®^ was developed. The aim of this single layer version was to enable a one-step procedure with the simultaneous application of autologous STSGs [95]. However, further studies are necessary to establish an Integra^®^ single layer in burn care.

Another option is the treatment with Matriderm^®^, a single layer dermal substitute, which consists of a collagen-based matrix and allows a one-step procedure in combination with an autologous split-thickness skin graft as an alternative for the missing epidermal layer [96,97]. The use of Matriderm^®^ in the treatments of burns in aesthetic and functional important areas achieved good results. Two studies by Ryssel et al. demonstrated an improved skin quality and range of motion in full-thickness burns of the hand’s dorsum treated with STSGs and Matriderm^®^ compared with ones treated by STSGs alone [98,99]. Furthermore, Matriderm^®^ is useful for the treatment of facial burns [96,100]. According to Jackson and Roman, the combination of Matriderm^®^ with split-thickness skin grafts is a safe and effective method to achieve better aesthetic and functional results in full-thickness facial burns [96].

Hyaluronic acid-based wound dressings were also introduced as an alternative for the dermal layer. Hyaluronic acid has a supportive role in the healing process, such as the stimulation of epidermal cell proliferation and migration, as well the promotion of fibroblast differentiation into myofibroblasts. Furthermore, hyaluronic acid improves the re-epithelization and granulation [101,102]. Hyalomatrix^®^ was designed as a temporary dressing in cases of deep burns and full-thickness wounds for wound bed preparation prior to definitive coverage with STSGs [103,104,105]. Faga et al. demonstrated that the application of STSGs followed by Hyalomatrix^®^ supports the dermis regeneration. The long-term biopsies showed that the regenerative skin was similar to healthy skin [106]. Gravante et al. even described that the aesthetic long-term result of patients treated with Hyalomatrix^®^ were similar to those treated with the combination of STSGs and Hyalomatrix^®^ [103].

In conclusion, the introduction of dermal substitutes led to burn care innovations, which enabled better functional and aesthetic outcomes. A major disadvantage is the dependence of the application of native skin. Future perspectives include the development of skin substitutes that are able to replace the dermal and the epidermal layer.

### 6.3. Cell Suspension

In 1895, Mangold described the first successful clinical application of scraped epithelial cells, which are known as a precursor of keratinocyte suspensions in modern burn care nowadays [107]. However, the technology was not able to be implemented due to the lack of an eligible carrier substance. Hunyadi et al. performed the first successful suspension of uncultured keratinocytes fixed on a fibrin carrier to treat chronic wounds in 1987 [108]. Nowadays, ReCell^®^ is a common method for the preparation of non-cultured autologous cells by the isolation of cells from a small donor site and immediate autologous replantation by spraying to promote the healing process. ReCell^®^ shows similar aesthetic results as STSGs and achieves an expansion ratio up to 1:80 [109,110,111]. The major benefit is the reduction of required donor sites for the treatment of high skin losses in severe burns [109,110,111]. However, the procedure is very time-consuming, which leads to greater surgical stress for the patients and additional surgery costs [110].

## 7. The Curse of Donor Site Morbidity

Donor site morbidity is a considerable problem in surgery burn care that has attracted attention in recent years. Despite the advantages of split-thickness skin grafting, the harvesting of donor sites creates secondary injuries. These injuries need wound care and can be associated with donor site morbidities such as pain, pruritus, wound infection, and hyperpigmentation as well unaesthetic and unpleasant hypertrophic scars [112,113]. While morbidities such as pain and scars are common, the infection rate seems to be low [113]. Almost no findings are available on the prevalence of hypertrophic scars associated with donor site [114,115]. According to retrospective analyses, 34% of reviewed patients were affected by persistent hypertrophic scarring [114]. Karlsson et al. demonstrated that 28% of the patients in a randomized longitudinal clinical trial had donor site hypertrophic scars [115]. However, scar formation seems to be a major problem for affected patients. In a cohort study, Legemate et al. evaluated the long-term scar quality of donor sites as stated by burn patients. Patients assessed the scar quality at 12 months after burn by using the Patient and Observer Scar Assessment Scale (POSAS) version 2.0. The patients’ overall opinion of the donor site scars was conspicuously high. The overall PSOAS score was 3.2 (1–10), even 1 year after surgery [116].

The usage of techniques, such as regrafting of the donor site or minced skin grafting, is described to reduce donor-site morbidity. Regrafting of the donor site is based on taking a larger amount of skin to cover not only the initial defect but also the donor site [117,118,119]. Several studies described an acceleration in re-epithelization, an improved scar quality, less pain, and a better aesthetic outcome by regrafting the donor site [117,118,119]. According to Bradow et al., all remaining pieces of a split-thickness skin graft should be placed back to the donor site. They also supposed that patients with poor healing potential could benefit from additional skin harvesting just for regrafting [117]. However, not all patients seem to benefit from the regrafting procedure. Legemate et al. demonstrated a worse result of the regrafted part compared with a non-grafted part in a follow up control of a 26-year-old woman 12 months after regrafting. While the non-grafted part was only a little erythematous, the regrafted part showed an irregular surface and a mix of hypo- and hyperpigmentation [120].

The technique of minced skin grafting is based on the use of the exceeded split skin, remaining after the application at the regular recipient area. These leftovers are prepared with tissue scissors until they get pasty enough to be dispensed onto the donor site [121,122]. Several studies showed a better quality of healing in terms of re-epithelialization and pigmentation, as well in a reduction of hypertrophic scarring and pruritus [121,122,123]. 

In conclusion, the application of STSG leftovers seems to be a promising way for the donor site treatment to reduce unpleasant morbidities. Especially, patients expected to develop a donor site morbidity could benefit from such procedures. Overall, a risk–benefit analysis could be useful to decide which patients could benefit by such procedures and who might not. Another technique to reduce donor site morbidity is dermal grafting, which is described below [124,125].

## 8. The Dermis Graft—A Novel Approach

Dermis grafting is a method to obtain a de-epithelialized split-thickness skin graft [124]. The technique of dermis grafting is based on the simultaneous harvesting of a purely dermal split-thickness graft from the same donor site after taking the standard split-thickness skin graft [124,125]. In this procedure, two grafts are obtained, and the dermis graft is always transferred to the recipient site, while the ordinary split-thickness skin graft can serve as an additional graft [125], or it can be used for donor site coverage [124]. Lindeford et al. observed no difference in the healing duration between the dermal grafts and standard split-thickness skin grafts [125]. According to Han et al., the dermis grafting is superior to the regular STSG technique not only due to the accelerated and improved healing of the donor site but also in terms of pigmentation, height, and vascularity at the recipient sites [124]. Altogether, dermal grafting is an interesting method, which enables obtaining two grafts from a single donor site to minimize the need of available skin in extended burns and to reduce donor site-associated morbidity.

## 9. Conclusions

Great achievements in the development of skin grafting were made, especially over the past 200 years. Many of them are still part of the current burn injury treatment. Nowadays, autologous split-thickness skin grafting is considered as the gold standard for the treatment of major traumatic loss of skin caused by burns. The development of expansion methods enables the coverage of large wound surfaces and increases the survival of severely burned patients. Additionally, to the survival of severe burns, the quality of survival, by preventing scars formation and contractures, is one of the main goals in burn injuries management. A more cosmetic and functional result can be obtained by the usage of full-thickness skin grafts in reconstruction. However, autologous skin grafting is limited by available donor sites, especially in the initial treatment of severely burned patients. Historically, various attempts were already made to reproduce the properties of healthy skin to fill this gap. Significant progresses were made in the development of skin substitutes, which are a great discovery and fulfill their purpose in the burn injuries treatment. However, commonly used skin substitutes are not able to achieve the properties of native skin. Almost all products are only able to replace one: the epidermal or dermal skin layers. As a current challenge, donor site morbidity, such as wound infection, hyperpigmentation, and hypertrophic scarring attract attention. Several approaches were already made to solve this problem. However, further investigations are needed. The long-term objective is the development of novel methods or combined techniques allowing covering large burn surfaces without the necessity of high amounts of donor sites. In summary, the desired result has not been achieved, and despite several drawbacks, autologous skin grafting remains the method of choice for burn coverage, even more than 3000 years after its discovery.

**Limitations:** This review has some inherent limitations. Many of the original articles are not written in the English language or are not available through the online database, which is caused by the fact that they are even older than the internet. Therefore, the content of this review is reliant on reprints and biographical literature. Finally, our review is limited to articles retrieved from PubMed and Google Scholar only with the possibility of missed publications.

## Data Availability

No new data were created or analyzed due to this study. Data sharing is not applicable to this article.
